# Advancements in the *in vitro* culture of human pluripotent stem cells: progress, challenges, and future directions: comprehensive review

**DOI:** 10.3389/ftox.2025.1667573

**Published:** 2025-12-02

**Authors:** Niraj Chaudhary, Luis G. Villa-Diaz

**Affiliations:** 1 Department of Biological Sciences, Oakland University, Rochester, MI, United States; 2 Department of Bioengineering, Oakland University, Rochester, MI, United States

**Keywords:** human pluripotent stem cells, feeder-free culture, xeno-free substrates, drug discovery, toxicology

## Abstract

The advancement of human pluripotent stem cell (hPSC) culture systems has revolutionized the landscape of preclinical drug discovery and toxicological evaluation. Progressing innovations from feeder-dependent and xenogeneic matrices to chemically defined, xeno-free, and fully synthetic platforms have addressed long-standing challenges in reproducibility, safety, and clinical translation. Developments in recombinant extracellular matrix proteins, synthetic peptide substrates, and polymer-based coatings have enabled the generation of Good Manufacturing Practice (GMP)-compliant, scalable hPSC cultures while minimizing biological variability and immunogenic risks. Integration of automation, artificial intelligence (AI), and three-dimensional (3D) bioprocessing technologies aims at further enhancement of standardization, quality control, and throughput. In the context of pharmaceutical research, hPSC-derived cellular models now underpin high-throughput drug screening and mechanistic toxicological assays, offering superior human relevance compared to traditional animal models. Despite these advances, barriers such as cellular immaturity, inter-batch variability, and limited regulatory acceptance persist, underscoring the need for further protocol standardization and technological refinement. This review provides a comprehensive analysis of current animal-free hPSC culture platforms, critically examines their strengths and limitations, and discusses future directions for advancing their application in drug discovery and predictive toxicology. The ongoing evolution of hPSC technologies promises to accelerate the development of safer, more effective therapeutic agents and to reshape the future of human disease modeling and pharmacological research.

## 1 Introduction: from the challenges to the potential of hPSC culture

### 1.1 hPSCs in the era of translational biomedicine

Human pluripotent stem cells (hPSCs) —including human embryonic stem cells (hESCs) derived from pre-implantation embryos (Thomson et al., 1998) and human induced pluripotent stem cells (hiPSCs) reprogrammed from somatic cells (Takahashi et al., 2007) are characterized by two fundamental capabilities: the ability to self-renew indefinitely *in vitro* while preserving pluripotency and the capacity to differentiate into virtually all specialized cell types of the human body, encompassing approximately 400 distinct lineages (Hatton et al., 2025). These attributes render hPSCs as essential tools for applications in regenerative medicine, drug development, disease modeling, and studies of human embryogenesis. To fully harness their therapeutic and research potential, it is critical to maintain hPSCs under defined culture conditions that support self-renewal and minimize spontaneous differentiation with minimal or no exposure to animal-derived products (Villa-Diaz et al., 2013).

Early hPSC culture methods utilized mouse embryonic fibroblast (MEF) feeder layers and fetal bovine serum-based media (Thomson et al., 1998). Subsequent advancements enabled feeder-free systems, incorporating Matrigel (Xu et al., 2001) and defined media such as mTeSR1 (Ludwig et al., 2006a). New developments brought in recombinant proteins such as vitronectin (Braam et al., 2008) and laminin-511/521 (Miyazaki et al., 2008), synthetic peptides such as Synthemax (Melkoumian et al., 2010) and StemAdhere (Nagaoka et al., 2010), and polymers like PMEDSAH (Villa-Diaz et al., 2010), advancing the development of fully synthetic substrates and three-dimensional culture systems optimized for large-scale expansion. Despite improvements, challenges remain, notably due to the risk of xenogeneic contamination and batch-to-batch variability associated with Matrigel and the substantial cost of defined culture components (Zhou et al., 2022).

Recent advances have focused on developing chemically defined, xeno-free culture systems to enable safe and efficient expansion of hPSCs. These defined conditions aim to eliminate variability and ensure scalability for large-scale production, crucial for clinical applications and research advancements. GMP for hPSCs ideally requires xeno-free, fully defined, and scalable culture systems and automated processes (Liu et al., 2020). Machine learning—often grouped under the broader umbrella of artificial intelligence (AI)—combined with automation can optimize hPSC culture, improving reproducibility and scalability for therapies and research. In addition, combining synthetic materials, including low-cost polymers and 3D configurations, with high-throughput processing compatible with pluripotency might create automated pluripotent stem cell factories (Park et al., 2023).

This review succinctly outlines the advancements in hPSC culture, highlighting the application of synthetic coatings as substrates to facilitate the indefinite proliferation of hPSCs *in vitro*.

### 1.2 Challenges in mechanistic investigations concerning feeder cells and xenogeneic elements

The incorporation of feeder cells and xenogeneic elements, including MEFs and matrices derived from animals, such as Matrigel, played a crucial role in the initial cultivation of hPSCs. Although these systems demonstrate the capacity of hPSCs for self-renewal and pluripotency, they also present considerable risks that obstruct mechanistic inquiries and translational applications (Table 1).

**TABLE 1:** Risks associated with feeder cells and xenogeneic components in hPSC culture.

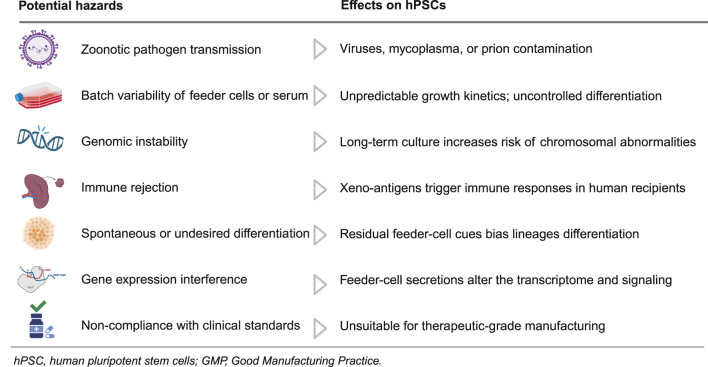

Table 1 summarizes the potential hazards of feeder cells and xenogeneic components in hPSC culture, detailing impacts on quality, safety, and translation. Feeder systems can introduce zoonotic contaminants, including viruses, *mycoplasma*, and prions (Cobo et al., 2005). This risk is compounded by batch-to-batch variability in feeders or serum that drives inconsistent proliferation kinetics and unscheduled differentiation, undermining assay reproducibility (Villa-Diaz et al., 2009; Ieyasu et al., 2017). Prolonged culture on animal-derived matrices further exacerbates instability by increasing chromosomal abnormalities and other genomic lesions (Mikkola et al., 2006), while incorporation of non-human sialic acids such as Neu5Gc heightens immunogenicity and the likelihood of rejection in human recipients (Martin et al., 2005). Persistent paracrine cues from feeder cells bias lineage specification, and undefined feeder-derived factors reshape transcriptomes and signaling networks, eroding phenotype fidelity (Shoji et al., 2018; Eiselleova et al., 2008; Wan et al., 2022). Because such xenogeneic systems are difficult to qualify under GMP, they remain unsuitable for therapeutic-grade manufacturing and regulated testing (Unger et al., 2008). Collectively, these considerations justify a transition to defined, xeno-free culture systems that support consistent expansion and translational testing of hPSCs.

#### 1.2.1 Feeder-free culture systems and their benefits in understanding the biology of hPSCs

The development of feeder-free culture systems marks a pivotal step toward standardizing hPSC cultivation and enhancing mechanistic studies of stem cell biology (Table 2). Unlike traditional feeder-dependent approaches, which rely on MEFs and animal-derived components, advanced feeder-free systems offer defined, xeno-free environments that reduce variability, eliminate cross-species contamination, and align with clinical manufacturing standards (Kawase and Nakatsuji, 2023). Although early feeder-free methods often used Matrigel (Xu et al., 2001), which is a complicated and inconsistent extract from mouse tumors (Hughes et al., 2010), advancements have resulted in the use of more reliable and clinically suitable materials, like recombinant laminin isoforms and synthetic polymer coatings (Villa-Diaz et al., 2013; Celiz et al., 2014a). These systems maintain hPSC self-renewal and pluripotency while providing a tractable platform for dissecting the molecular pathways that govern cell fate decisions. Consequently, feeder-free technologies not only support safe and scalable stem cell expansion for regenerative therapies but also enable reproducible, reductionist investigations into hPSC biology (Villa-Diaz et al., 2013; Kawase and Nakatsuji, 2023; Celiz et al., 2014a).

**TABLE 2:** Comparative evaluation of feeder-free vs. feeder-based culture systems in hPSC research.

Domain	Feeder-dependent	Feeder-free	Effect on hPSC culture	References
Safety/regulatory	• High xeno risk • Weak GMP fit	• Low xeno risk • Defined inputs	• ↑ Clinical suitability^#^	Villa-Diaz et al. (2009), Yuan et al. (2015), Dakhore et al. (2018), Ozawa et al. (2023), Soares et al. (2014), Englund et al. (2010)
Definition and reproducibility	• Undefined feeder secretome • Batch variability	• Defined media/matrices • High reproducibility	• ↓ Experimental noise^##^	Yuan et al. (2015), Villa-Diaz et al. (2009), Mei et al. (2010), James et al. (2005), Wang et al. (2005), Hanna et al. (2010)
Lot variability & QC burden	• Strong lot effects • Heavy QC	• Lower lot effects • Simpler QC	• ↓ Failure risk • ↓ Release burden	(Villa-Diaz et al., 2009; Celiz et al., 2014a; Yuan et al., 2015; Ozawa et al., 2023; Soares et al., 2014; Englund et al., 2010)
Scalability	• Labor-intensive prep • Hard to scale	• Large-format shown • Bioreactor-ready	• Supports high-volume work	Villa-Diaz et al. (2009), Dakhore et al. (2018), Ozawa et al. (2023), Soares et al. (2014), Schwedhelm et al. (2019)
Automation compatibility	• Manual co-culture • Manual passaging	• Robot/closed-system friendly • Inline monitoring	• Consistent, low-touch runs	Villa-Diaz et al. (2009), Yuan et al. (2015), Ozawa et al. (2023), Hanna et al. (2010)
Biological inputs	• Mixed endogenous cues • Hard to control	• Defined GF supplementation • Pluripotency maintained	• Clean perturbations	Villa-Diaz et al. (2009), James et al. (2005), Wang et al. (2005), Hanna et al. (2010)
Matrix/adhesion	• Feeder ECM provides adhesion	• Optimized coatings • Clonal attachment	• Adhesion signaling tuned	Villa-Diaz et al. (2009), Celiz et al. (2014a), Yuan et al. (2015), Englund et al. (2010)
Performance	• Often faster • Lot-to-lot variability	• Slightly slower • Predictable kinetics	• Plan passages/yields	Villa-Diaz et al. (2009), Celiz et al. (2014a), Yuan et al. (2015), Soares et al. (2014), Englund et al. (2010), Mei et al. (2010)
Economics	• Low reagents • High labor/footprint	• Higher reagents • Offset by scale/automation	• Better staff utilization	Yuan et al. (2015), Ozawa et al. (2023), Soares et al. (2014), Hanna et al. (2010)
Biological control	• Undefined factors confound	• Reductionist inputs • Easier causal inference	• Stronger mechanisms	Villa-Diaz et al. (2009), Yuan et al. (2015), Englund et al. (2010), Mei et al. (2010), James et al. (2005), Wang et al. (2005), Hanna et al. (2010)

# ↑ -maximizing, ## ↓-minimizing.

Feeder-free culture is superior for most hPSC expansion and differentiation, in addition to provide benefits in safety, reproducibility, scalability, automation, and mechanistic control (Yuan et al., 2015; Villa-Diaz et al., 2009; Dakhore et al., 2018; Ozawa et al., 2023; Soares et al., 2014; Englund et al., 2010; Mei et al., 2010; James et al., 2005; Wang et al., 2005; Hanna et al., 2010; Schwedhelm et al., 2019). Defined media and extracellular matrices lower xenogeneic inputs, align with translational workflows, and decrease experimental noise, whereas feeder layers introduce undefined factors that degrade reproducibility and drive lot variability (Yuan et al., 2015; Villa-Diaz et al., 2009; Dakhore et al., 2018; Ozawa et al., 2023; Soares et al., 2014; Englund et al., 2010; Mei et al., 2010; James et al., 2005; Wang et al., 2005). Targeted supplementation of core pathways (FGF2/TGF-β with BMP antagonism) stabilizes pluripotency and improves protocol transferability (Mei et al., 2010; James et al., 2005; Wang et al., 2005; Hanna et al., 2010). Operationally, feeder-free platforms reduce QC burden and support scale-up and automation, including real-time monitored aggregate workflows in stirred systems (Ozawa et al., 2023; Soares et al., 2014; Schwedhelm et al., 2019). Engineered adhesion substrates reproducibly replace feeder-derived ECM and support clonal growth (Yuan et al., 2015; Mei et al., 2010). Although feeders can yield faster proliferation, defined systems show more predictable kinetics and scheduling (Yuan et al., 2015; Villa-Diaz et al., 2009; Englund et al., 2010; Mei et al., 2010; James et al., 2005; Wang et al., 2005). In feeder-free conditions, costs shift toward reagents but are offset by reduced labor and scalable, weekend-light operations (Dakhore et al., 2018; Soares et al., 2014; Englund et al., 2010; Schwedhelm et al., 2019). Overall, feeder-free culture increases standardization, safety, and causal interpretability while sustaining pluripotency (Yuan et al., 2015; Ozawa et al., 2023; Soares et al., 2014; Mei et al., 2010; James et al., 2005; Wang et al., 2005; Hanna et al., 2010).

### 1.3 Redefining hPSC expansion: a journey from non-defined media to automated, AI-guided bioprocessing

hPSC have revolutionized regenerative medicine through their unique abilities to self-renew indefinitely and differentiate into all cell types. The evolution of hPSC culture methods represents a remarkable journey from traditional feeder-dependent systems to sophisticated automated platforms. As clinical applications demand scalable, standardized production, the field has progressed toward chemically defined conditions, synthetic substrates, and AI-guided bioprocessing, marking a transformative shift in stem cell manufacturing capabilities.

Building on this foundation, automation has emerged as a critical innovation to address the demands of consistent, large-scale hPSC production. Automation in stem cell culture has emerged as a pivotal advancement. Initial efforts, initiated over a decade ago, employed microcarrier-based and bioreactor platforms to support the large-scale expansion of neural progenitor cells (Sen et al., 2002). These systems offered important benefits, like high-throughput screening capabilities, consistent and reproducible culture conditions, and a reduced risk of microbial contamination (Liu et al., 2020). Nevertheless, early technologies encountered limitations such as elevated operational costs, uncontrolled cell aggregation leading to necrosis, and poor mechanical stability of culture materials like hydrogels (Kawase and Nakatsuji, 2023; Torizal et al., 2022). Recent advances have introduced chemically defined, xeno-free media and synthetic substrates to improve reproducibility, scalability, and GMP compliance (Rivera-Ordaz et al., 2021). Automated platforms now integrate high-throughput robotics, real-time monitoring, and standardized passaging (Schwedhelm et al., 2019; Tristan et al., 2021; Thomas et al., 2009). Closed-loop systems combining microfluidics and AI enable dynamic quality control, reducing manual variability and supporting scalable, clinically aligned biomanufacturing (Park et al., 2019).

Complementing automation, the incorporation of AI has further enhanced control and efficiency in stem cell bioprocessing. AI-guided bioprocessing in stem cell research emerged around 2014, introducing data-driven optimization to enhance the efficiency, reproducibility, and standardization of cell culture workflows (Joutsijoki et al., 2014). AI facilitates automated high-throughput screening, real-time monitoring, and decision-making algorithms that reduce human error and improve process consistency. Despite these advantages, challenges remain, including variability in experimental reproducibility and the need for standardized, high-quality training datasets. Current applications of AI in stem cell biology include predictive modeling in disease research, automated phenotyping, and the development of complex three-dimensional organoids (Park et al., 2019; Herland et al., 2020).

In parallel, advancements in three-dimensional (3D) culture have introduced more physiologically relevant platforms for hPSC expansion and differentiation. 3D culture systems for hPSC emerged in the mid-2000s as a strategy to more closely replicate the *in vivo* microenvironment (Mohr et al., 2006). These systems offer significant advantages, including enhanced scalability for large-scale expansion and improved nutrient and gas exchange, both critical for clinical translation. Nonetheless, several technical challenges, such as uncontrolled cell aggregation, which can lead to necrotic core formation, and difficulties in achieving uniform cell distribution are associated with 3D culture systems (Wu et al., 2014). Recent advancements have focused on engineering defined biomaterials, including peptide-based hydrogels and nanofibrous matrices, to provide structural support and improve culture homogeneity (Zhou et al., 2022).

Together, these emerging technologies converge to define the future landscape of stem cell biomanufacturing. In summary, the integration of 3D culture systems, automated bioprocessing, and AI is poised to transform large-scale hPSC manufacturing. Future directions will emphasize the combination of 3D platforms with bioreactor-based automation to enable robust, reproducible, and GMP-compliant production for regenerative and personalized therapies. AI, particularly when integrated with nanotechnology and 3D bioprinting, will enhance predictive modeling, adaptive control, and tissue engineering precision. These improvements are crucial for consistent hPSC production and improved reliability in applications such as disease modeling, drug screening, and clinical translation, marking a pivotal shift toward next-generation stem cell biomanufacturing.

## 2 Comparative analysis of animal-free culture systems for human pluripotent stem cells: approaches, challenges, and clinical implications

**FIGURE 1:**
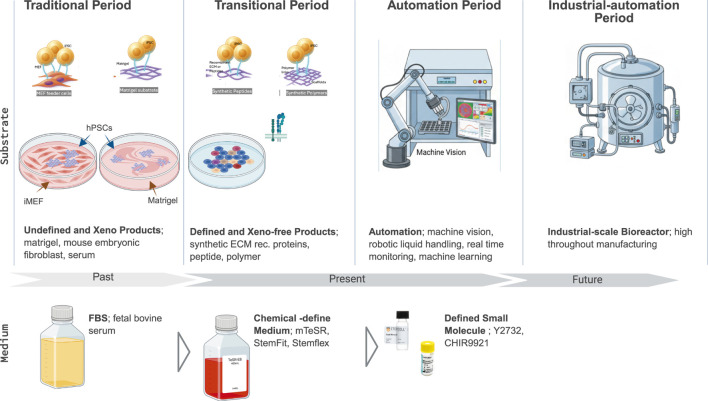
Comparative evolution of animal-free hPSC culture systems. Illustration showing the shift from feeder/xeno substrates and serum media to defined xeno-free matrices and media, then to robotic/AI-assisted automation, and finally industrial closed-bioreactor manufacturing.

hPSC expansion requires defined culture conditions to preserve self-renewal and pluripotency. Traditional systems use xenogeneic components such as feeder cells and Matrigel, introducing variability and risks of contamination. In response, xeno-free platforms using chemically defined media, recombinant human proteins, synthetic matrices, and plant-derived substrates (Liu et al., 2020) have been developed to enhance consistency, safety, and regulatory compliance. This section examines animal-free hPSC culture systems across the field’s evolution, spanning the past Traditional and Transitional periods, the current robotic, AI and machine-learning era and the future industrial-automated period aimed at scalable manufacturing (Figure 1).

### 2.1 Chemically defined and xeno-free media for animal-free hPSC culture

Chemically defined and xeno-free media represent a major advancement in hPSC culture, enabling consistent maintenance of pluripotency while eliminating the variability and risks associated with animal-derived components. Traditional systems such as mTeSR™1 (47), although feeder-free, contained bovine serum albumin and other xenogeneic additives. In response, next-generation formulations like Essential 8 (E8) (Chen et al., 2011) were developed to include only defined components—such as insulin, transferrin, FGF2, and TGF-β—while excluding serum albumin and β-mercaptoethanol to achieve full chemical definition. Further innovations have led to xeno-free commercial media, including TeSR-E8 (Chen et al., 2011), NutriStem hPSC XF (Biological Industries. Press Release, 2016), and StemFit® AK02N(50), which support GMP-compliant hPSC culture using recombinant or human-derived proteins on defined substrates (Skorik et al., 2020; Timilsina et al., 2023). Table 3 summarizes key features of these and other chemically defined, xeno-free media designed to support safe and reproducible hPSC culture in feeder-free conditions.

**TABLE 3:** Comparison of chemically defined and xeno-free media.

Culture media	Components	Xeno additives	Features	References
mTeSR™1 mTeSR Plus™	• FGF2 • TGF-β • Lithium • GABA • nutrients	BSA	• Early defined medium	Ludwig et al. (2006b)
Essential 8 (E8)	• Insulin • Transferrin • FGF2 • TGF-β • Nutrients • no BSA	None	• Fully defined • Xeno-free • albumin-free	Chen et al. (2011)
TeSR-E8	• Similar to E8	None	• Commercial variant of E8	Chen et al. (2011)
NutriStem XF	• Growth factor-reduced • recombinant proteins	None	• Xeno-free • Optimized for pluripotency	Biological Industries. Press Release (2016)
StemFit® AK02N	• Human albumin • Insulin • Transferrin • defined cytokines	None	• GMP-grade • Supports hiPSC expansion on laminin	Nakagawa et al. (2014)
Plant-derived media	• Plant- FGF2 • Plant- EGF • Plant- albumin	None	• Recombinant proteins • Produced in Oryza sativa cells	Lee et al. (2022)

Table 3 summarizes key features of these and other chemically defined, xeno-free media designed to support safe and reproducible hPSC culture in feeder-free conditions. The development of chemically defined, xeno-free media has significantly advanced hPSC culture by improving reproducibility, reducing contamination risks, and facilitating compliance with clinical manufacturing standards. Media formulations now commonly utilize recombinant human proteins or plant-derived analogs, such as plant-produced FGF2, EGF, and albumin, to achieve fully animal-origin-free systems (Lee et al., 2022). These innovations enable robust, scalable, and standardized platforms suitable for both research and therapeutic applications. Continued refinement of these systems will be essential for supporting the safe and efficient clinical translation of stem cell–based therapies.

### 2.2 Substrates for feeder-free culture

In addition to defined media, an animal-free hPSC culture system requires a suitable substrate or matrix for cell attachment. hPSCs survival and self-renewal are compromised by untreated tissue culture plastic and traditionally depend on either feeder cell layers or complex extracellular matrix (ECM) mixtures for adhesion and growth factor support.

#### 2.2.1 Matrigel as the gold standard substrate for iPSC culture: benefits and limitations

Matrigel has been the most used substrate for feeder-free culture of hPSC, including hiPSCs, due to its robust support for cell adhesion, proliferation, and maintenance of pluripotency (Xu et al., 2001). Derived from Engelbreth-Holm-Swarm (EHS) mouse sarcoma (Rodin et al., 2010; Kleinman et al., 1986), Matrigel is composed primarily of laminin, collagen IV, entactin, and perlecan, along with a complex mix of growth factors and over 1,800 unique proteins (Hughes et al., 2010). Despite its widespread utility in basic research, Matrigel’s undefined composition, xenogeneic origin, and batch-to-batch variability pose significant challenges for standardization and clinical translation (Dakhore et al., 2018; Ozawa et al., 2023). Although Matrigel remains a reliable matrix for experimental hPSC culture, its limitations have prompted a shift toward xeno-free, chemically defined, and GMP-compliant substrates for therapeutic applications (Melkoumian et al., 2010; Villa-Diaz et al., 2010). Advances in recombinant extracellular matrix proteins (e.g., laminin-511, vitronectin), synthetic peptides, and defined hydrogels offer improved reproducibility, safety, and scalability (Zhou et al., 2022; Ozawa et al., 2023; Miyazaki et al., 2012). These emerging platforms align with regulatory requirements and are more compatible with automation and bioreactor systems, facilitating the transition toward robust, clinical-grade iPSC expansion and differentiation (Celiz et al., 2014a).

#### 2.2.2 Synthetic and recombinant substrates for feeder-free culture

To eliminate the variability and xenogeneic risks associated with Matrigel and feeder cell systems, recombinant ECM proteins and fully synthetic substrates have been developed as defined alternatives for feeder-free hPSC culture.

Recombinant human ECM proteins have proven effective as defined substrates (Table 4). Laminins and vitronectin are two key adhesion ligands for hPSCs. Among these, vitronectin and fibronectin were among the first applied, with defined fragments like vitronectin-N supporting hPSC adhesion via αvβ5 integrins and maintaining self-renewal in xeno-free conditions (Braam et al., 2008). However, both vitronectin and fibronectin may show lower attachment efficiency or require higher coating concentrations compared to Matrigel (Badenes et al., 2016). In contrast, laminin-511 and laminin-521—basement membrane proteins expressed in the embryonic inner cell mass and placenta—more closely replicate the native hPSC niche (Xu et al., 2001). Laminin-511 supports long-term self-renewal (Rodin et al., 2010), while laminin-521 promotes single-cell survival (Rodin et al., 2014), clonal expansion, and sustained pluripotency. These substrates act primarily through α6β1 integrin signaling in hPSCs (Villa-Diaz et al., 2016) and are commercially available in defined, clinical-grade formats (e.g., iMatrix-511), making them widely used in GMP-compliant protocols for hiPSC expansion and directed differentiation.

**TABLE 4:** Recombinant human ECM protein substrates for hPSC maintenance.

ECM protein	Key components	Main advantages	Main limitations	References
Laminins	Laminin-511 (LN511)	• Support long-term self-renewal • Clonal derivation • α6β1 integrin engagement • Xeno-free • Defined	• Costly • Isoforms require optimization for specific applications	Rodin et al. (2010)
	Laminin-521 (LN521)			Rodin et al. (2014)
	Laminin-332 (LN332)			Miyazaki et al. (2008)
	Laminin E8 fragments			Miyazaki et al. (2012)
Recombinant Vitronectin	Truncated VTN-N, Full-length recombinant vitronectin	• Defined • Xeno-free • Supports pluripotency via αvβ5 integrin	• Batch quality variation • Limited support for certain hPSC lines	(Braam et al., 2008; Chen et al., 2011)
E-cadherin	Recombinant E-cadherin (combined with LN521)	• Enhances clonal derivation • Survival in defined conditions	• Limited adhesion efficiency alone • Often used in combination	Nagaoka et al. (2010)

On the other hand, synthetic peptide-based substrates have emerged as defined and xeno-free alternatives to traditional extracellular matrix coatings for hPSC culture (Table 5). Vitronectin-derived peptides such as VN1:KGGPQVTRGDVFTMP (Deng et al., 2013), VN2: CGGKKQRFRHRNRKG (Park et al., 2015), and commercially available Synthemax (a vitronectin peptide–acrylate conjugate) (Melkoumian et al., 2010) support robust cell adhesion, self-renewal, and long-term maintenance of pluripotency under feeder-free conditions. These substrates are scalable and chemically defined, making them well-suited for research and preclinical applications (Badenes et al., 2014). However, their performance may vary across different hPSC lines and often requires optimization. RGD-containing peptides, including heparin-binding variants GKKQRFRHRNRKG (Klim et al., 2010) and optimized vitronectin mimetics (e.g., Ac-KGGPQVTRGDTYRAY) (Zhou et al., 2018) function by mimicking natural integrin-binding domains to promote cell attachment and survival. While promising for translational use, these systems are sensitive to peptide density and conformation, which may affect reproducibility. Overall, synthetic peptide substrates represent a critical step toward standardized, xeno-free platforms for hPSC expansion and differentiation.

**TABLE 5:** Synthetic peptide-based culture systems for hPSC maintenance.

Peptide	Key components	Details	Mechanism	Advantages limitations	Ref.
Vitronectin-Derived Peptides	VN1: KGGPQVTRGDVFTMP	• Supports hPSC adhesion on synthetic hydrogels/films	• VN RGD-region peptide with N-terminal acetylated	• Xeno-free • Defined • Supports adhesion and self-renewal • Scalable • Cell-line specific optimization needed	Deng et al. (2013)
	VN1: CGGKKQRFRHRNRKG	• Superior attachment due to disulfide-mediated dimerization	• VN heparin-binding motif with Cys-GlyGly handle		Park et al. (2015)
Synthemax	Vitronectin, acrylate polymer	• Peptide-acrylate surface	• Vitronectin-derived adhesive peptides on an acrylate polymer	• Used as a Matrigel replacement in downstream differentiation	Melkoumian et al. (2010)
RGD-Containing Peptides	GKKQRFRHRNRKG	• Vitronectin heparin-binding peptide	• Glycosaminoglycan-binding substratum	• Supports hPSC VN RGD peptide	Klim et al. (2010)
	Ac-KGGPQVTRGDTYRAY	• Engineered RGD peptide	• Designed by modifying residues flanking RGD		Zhou et al. (2018)

Synthetic polymer-based substrates have been developed as defined, xeno-free platforms for the maintenance and expansion of hPSCs (Table 6). Polyzwitterion, acrylate, and methacrylate polymers such as PMEDSAH (Villa-Diaz et al., 2010), APMAAm (Higuchi et al., 2015), and Poly (HPhMA-co-HEMA) (Deng et al., 2013) support long-term self-renewal and pluripotency in feeder-free conditions, although their synthesis and surface coating protocols can be complex. Other synthetic polymers, including Poly (TCDMDA-blend-BA) (Park et al., 2015), PMVE-alt-MA (Chen et al., 2017), and, PVB (Otsuji et al., 2014) offer biocompatibility and GMP potential; however, they often require cell-type-specific optimization. Temperature-responsive polymers like PEGMA brush (Celiz et al., 2014b), PVA (Forghani et al., 2017), and PNIPAM (Otsuji et al., 2014) enable enzyme-free passaging and are promising for automation and large-scale production; however, they are still in the experimental stage due to concerns regarding stability and reproducibility. Collectively, these materials contribute to the development of scalable, standardized hPSC culture systems compatible with clinical applications.

**TABLE 6:** Synthetic polymer-based culture systems for hPSC maintenance.

Polymer	Year	Key components	Details	Advantages limitations	Ref.
Polyzwitterion (Methacrylate) coatings					
PMEDSAH (Surface coating polymer)	2010	Zwitterionic methacrylate monomer	Zwitterion mimic the function of heparin, support PSC adhesion	• Fully synthetic • Supports long-term culture • Requires pre-conditioning with serum/media	Villa-Diaz et al. (2010)
Acrylate substrates					
Peptide-acrylate surface (PAS/Synthemax)	2010	Acrylate polymer with VN-peptide tether	Presented VTN-derived RGD motif directly engages αVβ5 (±α5β1) integrins	• Chemically defined • Xeno-free • Expensive • Induces spontaneous differentiation	Melkoumian et al. (2010)
Thermo-responsive acrylate hydrogel	2013	Copolymers of 2-(diethylamino)ethyl acrylate with AEtMA-Cl	Protein adsorption–mediated integrin adhesion on cationic/anionic copolymer Temperature-driven swelling reduces adhesion for gentle detachment	• Gentle enzyme-free cell detachment • Slower cell growth • Chromosomal abnormalities	Forghani et al. (2017)
Poly (TCDMDA-blend-BA)	2021	Tricyclodecane dimethanol diacrylate + butyl acrylate	Protein-adsorption layer enriches TGF-β1; αVβ3/αVβ5-mediated adhesion	• Scalable • Low cost • Slow growth rate	Nasir et al. (2021)
Methacrylamide substrates					
APMAAm	2011	aminopropylmethacrylamide hydrogel	BSA from medium to facilitate cell attachment	• Supports long-term culture • Requires protein adsorption from media	Irwin et al. (2011)
PEGMA brush	2013	Oligo ethylene glycol methacrylate (OEGMA) + 2-hydroxyethyl methacrylate (HEMA)	Covalently grafted VN-peptide provides stable integrin ligands on antifouling brush	• Does not require protein pre-conditioning • Tunable • Requires grafting chemistry	Deng et al. (2013)
TCDMDA-blend-BA	2015	N-(4-hydroxyphenyl) methacrylamide + HEMA	Protein-adsorption–mediated αV/β1-integrin engagement	• Xeno-free • Discovery platform entry	Celiz et al. (2014b)
pGC2	2024	Poly (glycidyl methacrylate-g-guanidine-co-carboxylic acrylate)	Mixed guanidine and carboxylate groups modulate protein adsorption and cell–surface interactions enabling integrin-dependent adhesion	• Supports long-term culture • Requires protein adsorption from media	Cho et al. (2024)
Polyvinyl substrates					
PMVE-alt-MA	2010	Poly (methyl vinyl ether-alt-maleic anhydride)	Protein adsorption enables integrin-mediated attachment	• Supports long-term hPSC self-renewal • Cost-effective • Requires controlled coating	Brafman et al. (2010)
PVA hydrogels	2017	Polyvinyl alcohol-co-itaconic acid; VN-derived peptides via PEG linkers	Peptide-mediated integrin adhesion on bioinert PVA base	• Defined • Tunable • Requires peptide conjugation	Chen et al. (2017)
PNIPAM dishes	2019	Poly (N-isopropylacrylamide) coatings/dishes	LCST switch changes surface wettability to release sheets	• Gentle passaging • Substrate stability • Enzyme-free detachment	Cho et al. (2024)
PVB (plastic scaffold)	2022	Polyvinyl butyral + integrin-binding peptide	Peptide-driven integrin adhesion on PVB	• Easy coating • Storage-stable • Peptide sourcing needed	Otsuji et al. (2014)

In summary, the development of synthetic and recombinant substrates has markedly improved feeder-free culture systems for hPSCs by introducing defined, xeno-free, and scalable alternatives to biologically derived matrices. Although further work is needed to address issues related to cost-efficiency, cell-line-specific optimization, and regulatory validation, these substrates represent a pivotal advancement toward the establishment of standardized, GMP-compliant platforms for clinical-grade stem cell manufacturing.

### 2.3 Three-dimensional (3D) culture systems

Three-dimensional (3D) culture systems have emerged as essential platforms for hPSC maintenance and expansion, offering improved physiological relevance compared to traditional two-dimensional systems. By facilitating enhanced cell–cell and cell–matrix interactions, 3D cultures better replicate *in vivo* conditions and enable scalable production required for downstream clinical and research applications. Common 3D strategies include aggregate cultures (scaffold-free and scaffold-supported), microwell/ULA plate–based aggregation, microcarrier-based suspension systems, microencapsulation in hydrogels, bio-printed constructs, acoustic/levitation assembly, and organ-on-chip (microfluidic) platforms, each with distinct biochemical and mechanical properties (Zhou et al., 2022). These approaches have been adapted for feeder-free and xeno-free culture, aligning with regulatory and GMP standards. Table 7 outlines the key components, advantages, and limitations of the major 3D hPSC culture systems currently used in the field.

**TABLE 7:** Three-dimensional (3D) culture systems for hPSC maintenance.

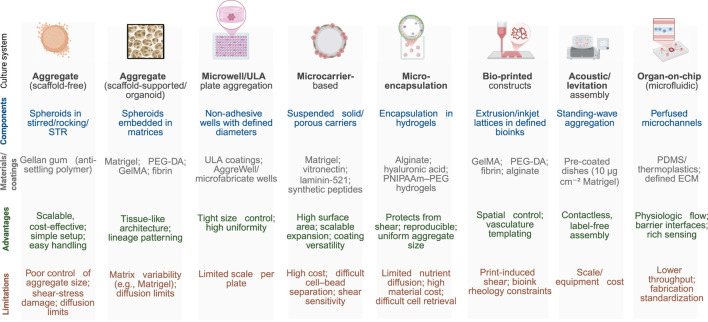

As summarized in Table 7, each 3D culture system has specific strengths and limitations. Aggregate methods are cost-effective and easy to implement but may suffer from poor size control and shear stress-related damage (Otsuji et al., 2014; Lancaster et al., 2013). Microwell/ULA plate systems provide tight size control and high uniformity but are constrained by plate scale and throughput (Mohr et al., 2010). On the other hand, scaffold-supported aggregates embed spheroids in defined hydrogels (e.g., PEG-DA, GelMA, fibrin), adding tissue-like architecture but inheriting matrix variability and diffusion limits. Microcarrier-based systems also offer high surface area for large-scale expansion, with common coatings such as vitronectin, laminin-521, or synthetic peptides; however, challenges associated include coating cost, cell–bead separation, and shear sensitivity (Oh et al., 2009). Microencapsulation protects cells and standardizes aggregate size using alginate, hyaluronic acid, or PNIPAAm–PEG hydrogels, yet is limited by nutrient diffusion, difficult cell retrieval, and material costs (Kim et al., 2013). Bio-printed constructs enable spatial control and vasculature templating in printable bioinks (GelMA, PEGDA, fibrin, alginate), however, face print-induced shear and rheology constraints (Faulkner-Jones et al., 2015). Acoustic/levitation assembly is contactless and label-free, but requires specialized equipment and currently can be achieved in modest scale (Hahn et al., 2025). Organ-on-chip (microfluidic) platforms recreate barrier interfaces and perfusion on PDMS or thermoplastics with defined ECM coatings, trading physiological fidelity for lower throughput and fabrication standardization (Ellis et al., 2017). Thus, the choice of system should be guided by application-specific needs—such as scalability, reproducibility, and clinical compatibility—as the field advances toward standardized, GMP-compliant hPSC manufacturing.

### 2.4 Recent advances in automating the techniques for culturing hPSCs

Reprogramming hiPSCs is a multi-step, time-consuming process requiring strict sterility and continuous manual monitoring. This process is inherently labor-intensive and prone to variability. Cellular heterogeneity resulting from lineage reprogramming complicates the isolation of monoclonal hiPSC lines. These challenges underscore the need for scalable, standardized cell manufacturing platforms to enable efficient clinical and research applications (Celiz et al., 2014a). Manual culture techniques are incompatible with high-throughput production, driving the demand for automation; therefore, advances in automated technologies have led to the development of several platforms for hiPSC reprogramming and maintenance.

Table 8 summarizes key technological advancements in automating the generation and cultivation of hiPSCs. It highlights pre-automation and early machine-vision monitoring for continuous, noninvasive observation (Narkilahti et al., 2007), the first bench-scale liquid-handling platforms for standardized maintenance and differentiation (Thomas et al., 2009), automated imaging with colony picking to improve clonality (Haupt et al., 2012), machine-learning quality control based on colony and nuclear morphology (Tokunaga et al., 2014), deep-learning segmentation enabling objective masks (Zhang et al., 2019), and time-lapse AI evaluation for label-free QC (Kato et al., 2016), CNN-based colony classification for rapid triage (Witmer and Bhanu, 2018), and modular robotics for closed, GMP-oriented manufacturing (Tissue Factory, StemCellFactory, and high-throughput robotic lines) (Tristan et al., 2021; Kikuchi et al., 2018; Zhang et al., 2019; Elanzew et al., 2020). Recent U-Net–guided clone picking further reduces operator bias while improving clonality (Powell et al., 2023). Together, these systems improve reproducibility, reduce variability, and support GMP-compatible scaling from reprogramming through expansion and directed differentiation.

**TABLE 8:** Key technological advancements in automating the generation and cultivation of hiPSC.

Automation	Key technologies	Advantages	Limitations	Year	Ref.
Machine vision system	Noninvasive time-lapse monitoring of living cultures	• Continuous • Label-free monitoring	• Limited analytics in early work	2007	Narkilahti et al. (2007)
Pre-automated PSC culture Platform	Robotic feeder-free hESC expansion	• Reproducible • Hands-off passaging	• Platform rigidity • Capital cost	2009	Thomas et al. (2009)
Automated imaging and colony selection	Image-guided colony detection and robotic picking	• Precise, gentle colony isolation	• Throughput • Device cost	2012	Haupt et al. (2012)
Machine-learning culture Platform	Supervised ML to distinguish *bona fide* iPSCs	• Noninvasive QC • Scalable scoring	• Generalization across labs	2014	Tokunaga et al. (2014)
Deep learning + U-Net	Semantic segmentation of colony images	• Accurate/scalable segmentation for automation	• Not PSC-specific • Needs labeled data	2015	Ronneberger et al. (2015)
Time-lapse imaging + AI evaluation	Automated analysis of time-lapse morphology for QC	• Label-free metrics • Objective thresholds	• Imaging burden • Feature engineering	2016	Kato et al. (2016)
hESCnet	CNN classifier for hESC colonies	• High classification accuracy with limited real data	• Does not measure pluripotency markers directly	2018	Witmer and Bhanu (2018)
Flexible modular “Tissue Factory”	Modular robotic biomanufacturing platform	• GMP-compatible • Multi-lineage production	• Complex integration • High infrastructure cost	2018	Kikuchi et al. (2018)
Progenitor cell identification platform	ML on time-lapse to identify iPSC progenitors	• Earlier selection • Reduces waste	• Modest precision • Model-specific	2019	Zhang et al. (2019)
StemCellFactory robotic platform	End-to-end robotic hiPSC generation and expansion	• Integrated • Feeder-free • Scalable	• Custom setup • Tech lock-in	2020	Elanzew et al. (2020)
Robotic biomanufacturing (GMP-oriented)	High-throughput hiPSC maintenance and differentiation	• Throughput • Standardized protocols	• Facility-specific automation	2021	Tristan et al. (2021)
Cell X™ robotic system	U-Net–guided colony segmentation and robotic picking	• Improves colony purity and clonality	• Relies on accurate annotation and model training	2023	Powell et al. (2023)

### 2.5 Validation of pluripotency under animal-free conditions

A key concern in adopting animal-free hPSC culture systems is whether they can robustly support self-renewal and multilineage differentiation. Multiple studies have demonstrated that hPSCs maintained under feeder-free, xeno-free conditions retain hallmark pluripotency markers—such as OCT4, NANOG, SSEA4, and TRA-1-60—in over 90%–95% of cells, as confirmed by immunostaining and flow cytometry (Villa-Diaz et al., 2010; Badenes et al., 2014; Shimizu et al., 2022). These cultures also preserve a stable diploid karyotype across extended passages in defined media like Essential 8 and TeSR-E8. Furthermore, gene expression analyses show sustained activation of core pluripotency genes (e.g., SOX2, OCT4, NANOG), confirming genomic stability and undifferentiated status (Timilsina et al., 2023). Critically, hPSCs expanded under animal-free conditions retain full developmental potential, as evidenced by their ability to differentiate into all three germ layers *in vitro* and *in vivo*, including through embryoid body and teratoma assays (Villa-Diaz et al., 2010; Chen et al., 2017). Collectively, these findings validate that animal-free culture systems are suitable for maintaining both molecular and functional pluripotency, supporting their use in clinical and translational applications.

### 2.6 Clinical and translational implications of animal-free hPSC culture

The transition to animal-free hPSC culture is driven by the increasing demand for regulatory compliance and clinical-grade manufacturing. Regulatory agencies such as the U.S. Food and Drug Administration (FDA), European Medicines Agency (EMA), and Japan’s Pharmaceuticals and Medical Devices Agency (PMDA) mandate the use of xeno-free, traceable, and well-characterized reagents to mitigate risks associated with animal-derived components (Ozawa et al., 2023; Tanaka et al., 2023). In alignment with current GMP standards, defined and animal-free systems— such as Essential 8 medium and recombinant laminins— are now integral to the production of clinical-grade hPSC derivatives. These platforms have already supported early-phase clinical trials, including transplantation of hESC-derived retinal pigment epithelial (RPE) cells for macular degeneration without culture-related adverse events (Da Cruz et al., 2018). Beyond safety, animal-free systems offer enhanced scalability and integration with bioreactor-based bioprocesses. Unlike feeder-dependent systems or Matrigel, defined synthetic substrates and microcarriers can be incorporated into automated workflows for large-scale stem cell production. Demonstrations of successful hPSC expansion in stirred-tank bioreactors using xeno-free media underscore the translational feasibility of these systems (Krawetz et al., 2010). Furthermore, regulatory submissions now require comprehensive Chemistry, Manufacturing, and Controls (CMC) documentation, including the provenance of all culture components. In addition, animal-free systems simplify CMC dossiers by minimizing immunogenic risks and offering consistent manufacturing inputs (Herland et al., 2020). The 2024 FDA guidance further mandates full disclosure and justification of all culture materials in Investigational New Drug (IND) applications, positioning recombinant human albumin and other synthetic substitutes as preferred alternatives. Collectively, these advances ensure that hPSCs cultured under animal-free conditions can meet the stringent quality, safety, and scalability requirements of regenerative therapies.

### 2.7 Toward safe, scalable, and regulatory-compliant hPSC culture systems

The transition to animal-free, chemically defined culture systems marks a pivotal evolution in hPSC research, offering enhanced consistency, safety, and regulatory readiness. As demonstrated across various platforms—from xeno-free media and synthetic substrates to three-dimensional bioreactor-compatible matrices—these systems maintain the core properties of pluripotency while enabling scalable and GMP-compliant manufacturing. Comparative analysis reveals that while each system has distinct advantages and limitations, collectively they represent a robust foundation for clinical grade hPSC expansion and differentiation. Ongoing validation of genomic stability, lineage specificity, and therapeutic efficacy will be essential to fully realize the translational potential of these innovations. Ultimately, the adoption of animal-free systems is not only a technical refinement but also a necessary step toward ethical, standardized, and clinically viable stem cell-based therapies.

## 3 hiPSC technology in drug discovery and regenerative medicine

hiPSC technology has advanced the clinical and pharmaceutical research landscapes. By generating human disease models with unprecedented accuracy, hiPSCs facilitate in-depth clinical investigations that were previously unattainable. In pharmaceutical research, hiPSC-derived models enable high-throughput screening and toxicity studies, revolutionizing drug discovery by accelerating the identification of potential therapeutics. In the future, hiPSC technology is expected to enable clinical applications—including tissue and organ replacement and personalized medicine via disease-in-a-dish models—providing new avenues for tailored treatments and improved patient outcomes. This convergence of scientific innovation holds immense promise for reshaping medical research paradigms and improving healthcare delivery worldwide.

### 3.1 hiPSC-based disease modeling

hiPSC-based disease modeling has revolutionized biomedical research by enabling the generation of patient-specific cells that retain the genetic background of the donor. Since their introduction in 2006, hiPSCs have provided a renewable source of pluripotent cells capable of differentiating into disease-relevant lineages, thereby allowing mechanistic studies in a human genetic context (Takahashi et al., 2007; Vo et al., 2024). Compared to hESCs, hiPSCs bypass ethical concerns and are more accessible, facilitating the study of a broader range of inherited and sporadic diseases. The integration of genome editing tools such as CRISPR/Cas9 further enhances model fidelity by enabling the generation of isogenic controls, which help distinguish pathogenic variants from background genetic noise (Hockemeyer and Jaenisch, 2016). Together, these capabilities position hiPSCs as a powerful system for modeling complex human diseases with unprecedented precision.

As summarized in Tables 9–11, diverse organoid systems have been established for diseases of endodermal, mesodermal, and ectodermal origins, including lung, liver, gastric, intestinal, pancreatic organoids, cardiac, renal-nephrotic, muscular organoids/spheroid, brain, cerebral, retinal, spinal organoids. In addition to organoids following are examples of popular disease modeling: ARPKD disease models, Polycystic Kidney Disease, Seckel syndrome, Miller-Dieker lissencephaly, Alzheimer’s disease, Fragile X syndrome, Leigh syndrome, Parkinson’s disease, Rett syndrome, FTD, Spinal muscular atrophy, Glaucoma, RP, FRD, altogether demonstrating the versatility and precision of this modeling approach.

**TABLE 9:** Overview of key hPSC-derived organoids in disease research and modeling with endoderm origin.

Cell source	Disease modeled	Organoid	Phenotype	References
Lung organoids				
hiPSCs	Cystic fibrosis (CF)	Proximal airway organoids	Reduced forskolin-induced swelling	McCauley et al. (2017)
hiPSCs	Cystic fibrosis	Proximal airway organoids	Smaller steady-state lumen area and reduced forskolin-induced swelling	Demchenko et al. (2023)
hESCs	Hermansky-Pudlak syndrome (HPS)	Lung organoids	Pulmonary fibrosis	Strikoudis et al. (2019)
hiPSCs	Inherited deficiency of surfactant protein B	Lung organoids	Abnormal lamellar body formation	Leibel et al. (2019)
hiPSCs	Surfactant deficiency	Type 2 Epithelial cells	Diminished surfactant production	Jacob et al. (2017)
Liver Organoids				
hiPSCs	Alagille syndrome	Cholangiocytes	Impaired organoid formation	Sampaziotis et al. (2015)
hiPSCs	ARPKD	Cholangiocytes	Dysfunctional epithelial transport	Ogawa et al. (2021)
hiPSCs	Autosomal-recessive polycystic kidney disease	Hepatic organoids	Abnormal bile ducts and fibrosis	Guan et al. (2021)
Gastric Organoids				
hiPSCs	*Helicobacter Pylori* infection	Epithelial and neuroendocrine	Increased epithelial proliferation	McCracken et al. (2014)
Intestinal Organoids				
hiPSCs	Hirschsprung disease	epithelial and enteric nervous system	Impaired enteric nervous system organization	Workman et al. (2017)
hiPSCs	Colorectal cancer with polyposis	Colonic organoids	Enhanced WNT activity and increased cell proliferation	Crespo et al. (2017)
hiPSCs	Colon cancer	Colonocytes	Epithelial proliferation	Sommer et al. (2018)
Pancreatic Organoids				
hESCs	Pancreatic ductal adenocarcinoma	Exocrine progenitor organoids	Mortality *in vivo* after transplantation	Breunig et al. (2021)
hiPSCs, hESCs	Pancreatic ductal adenocarcinoma	Pancreatic duct-like organoids	Intraductal papillary mucinous neoplasia-like structures	Hohwieler et al. (2017)
hiPSCs	Biliary development	Cystic organoids	Studying biliary development	Hohwieler et al. (2017)

**TABLE 10:** Overview of key hPSC-derived organoids in disease research and modeling of mesoderm origin.

Cell Source	Disease modeled	Organoid	Phenotype	References
hiPSCs	Regeneration	Cardiomyocytes	• Fetal-like cardiomyocyte	Mills et al. (2017)
hiPSCs	Angiomyolipoma (AML)	Renal organoids	• Myomelanocytic cells • Epithelial cysts	Hernandez et al. (2021)
hiPSCs, hESCs	Tuberous sclerosis	Renal organoids	• Angiomyolipoma • Myomelanocytic phenotype	Pietrobon et al. (2022)
hiPSCs	Nephrotic syndrome	Kidney organoids	• Hypertrophied podocyte bodies	Tanigawa et al. (2018)
hiPSCs	Nephrotic syndrome	Kidney organoids	• Decreased tuft formation • Increased apoptosis	Majmundar et al. (2021)
hiPSCs, hESCs	Polycystic kidney disease	Kidney organoids	• Ciliopathy phenotypes • Lack of cilia	Kuraoka et al. (2020)
hiPSCs	ARPKD	Organoid-on-a-chip	• Distal nephron cyst formation	Tran et al. (2022)
hiPSCs	Amyotrophic lateral sclerosis	Motor-neuron spheroids on a chip	• Decreased muscle contractions • Degradation of motor neurons • Increased apoptosis in muscles	Osaki et al. (2018)
hiPSCs	Muscular dystrophies	Multilineage muscle	• Nuclear elongation	Maffioletti et al. (2018)

Phenotypic characteristics highlighted in red font indicate a deficiency in the organoid model.

**TABLE 11:** Overview of key hPSC-derived organoids in disease research and modeling of ectoderm origin.

Cell source	Disease modeled	Organoid	Phenotype (modeling)	References
Brain organoids				
hiPSCs	Autism spectrum disorders	Brain organoids	• Autism spectrum disorders	Mariani et al. (2015)
hESCs, hiPSCs	Zika virus infection	Brain organoids	• Zika virus infection	Garcez et al. (2016)
hiPSCs	Zika virus infection	Brain-region-specific organoids	• Zika virus infection • Brain Development	Gabriel et al. (2016)
hiPSCs	Seckel syndrome	Brain organoids	• Seckel syndrome	Ichisima et al. (2019)
hESCs, hiPSCs	Species difference	Cortical organoids	• Modeling species difference	Kanton et al. (2019)
hiPSCs	Miller-Dieker lissencephaly	NESCs	• Smaller organoids • Apoptosis	Bershteyn et al. (2017)
hiPSCs	Alzheimer’s disease (AD)	Cerebral organoids	• Accelerated neural differentiation	Meyer et al. (2019)
hiPSCs	Fragile X syndrome (FXS)	Forebrain organoids	• Dysregulated neuronal development	Kang et al. (2021)
hiPSCs	Leigh syndrome (LS)	Cortical brain organoids	• Neural morphogenesis	Romero-Morales et al. (2022)
Cerebral organoids				
hiPSCs	Alzheimer’s disease (AD)	Cerebral organoids	• Exacerbated tau pathology	Zhao et al. (2020)
hESCs	Parkinson’s disease	Midbrain organoids	• Impaired midbrain-type dopamine neuron development via impaired WNT-LMX1A signaling	Wulansari et al. (2021)
hiPSCs	Microcephaly	Neurons, NPCs, RG, Retina, Choroid Plexus, Meninges	• Premature neuronal differentiation	Lancaster et al. (2013)
hESCs, hiPSCs	Microcephaly	Cerebral organoids	• Modeling human cortical development and microcephaly	Pellegrini et al. (2020)
hESCs, hiPSCs	Neocortex development	Cerebral organoids	• Modeling human fetal neocortex development	Kadoshima et al. (2013)
hiPSCs	Seckel syndrome microcephaly	Neurons, RG	• Smaller organoids • Premature neuronal differentiation	Gabriel et al. (2016)
hiPSCs	Rett syndrome	Neurons, NPCs	• Increased ventricular area • Impaired neurogenesis	Mellios et al. (2018)
hiPSCs	Glioblastoma	NPCs	• Organoid overgrowth	Bian et al. (2018)
hiPSCs	Frontotemporal dementia (FTD)	Cerebral organoids	• Increased susceptibility to glutamate toxicity	Bowles et al. (2021)
Spinal organoids				
hiPSCs	Spinal muscular atrophy	Spinal organoids	• Motor-neuron degeneration	Hor et al. (2018)
hiPSCs	Mitochondrial encephalomyopathy, lactic acidosis, and stroke-like episodes	Spinal-cord organoids	• Motor neurons	Winanto et al. (2020)
Retinal organoids				
hiPSCs	Glaucoma	Retinal organoids	• Glaucoma	VanderWall et al. (2020)
hiPSCs	Retinitis pigmentosa (RP)	Retinal epithelium and organoids	• Photoreceptor number • Cilia length	Rodrigues et al. (2022)
hiPSCs	Retinitis pigmentosa	Retinal organoids	• Photoreceptor degeneration	Lane et al. (2020)
Other organoids				
hiPSCs	Cortical development and diseases	Cortical spheroids	• Cortical development and diseases	Paşca et al. (2015)
hiPSCs	Friedreich’s ataxia (FRDA)	Dorsal-root-ganglia organoids	• Impairment in axonal spreading	Mazzara et al. (2020)

### 3.2 hiPSC-based drug discovery and development

hiPSCs have transformed drug discovery by enabling high-throughput screening (HTS) platforms that are both physiologically relevant and scalable. By differentiating into disease-relevant cell types, hiPSCs support mechanistic studies, phenotypic screening, and safety profiling across diverse disease models.

Table 12 summarizes hiPSC-based high-throughput screening strategies across platforms, assay modalities, and applications. Isogenic disease modeling uses genome-edited pairs to isolate genotype–phenotype relationships and quantify drug responses (Soldner et al., 2011). High-content imaging enables automated, scalable morphology and multiparameter phenotyping (Bray et al., 2016). Electrophysiological assays, including microelectrode arrays and calcium imaging, measure neural and cardiac function and detect proarrhythmic or neurotoxic effects (Sala et al., 2018). Chemogenomic phenotypic screening applies annotated compound libraries to map pathway dependencies and support target validation (Grafton et al., 2021). Organotypic co-cultures—organoids and microfluidic systems—reconstitute tissue architecture and cell–cell interactions for context-aware screening (Huh et al., 2010). Multi-omics integration aggregates transcriptomic and proteomic readouts to discover biomarkers and stratify patient-relevant responses (Volpato et al., 2018). Together, these modalities provide quantitative phenotypes, strengthen mechanism-of-action inference, and increase translational value for preclinical decision-making.

**TABLE 12:** Summary of iPSC-based high-throughput screening (HTS) strategies and their applications.

Strategy	Description	Key applications	Ref.
Disease Modeling & Isogenic Controls	Genome-edited iPSCs to generate isogenic disease models enabling controlled genotype-phenotype analyses	• Rare disease modeling • Drug efficacy • Toxicity prediction	Soldner et al. (2011)
High-Content Imaging & Morphometry	Automated imaging and morphometric analysis to quantify disease phenotypes and screen compound libraries	• Neurodegenerative disease phenotyping • Synaptic assays	Bray et al. (2016)
Electrophysiological Platforms	MEA and calcium imaging to assess electrophysiological responses in iPSC-derived neurons and cardiomyocytes	• Cardiotoxicity screening • Neuronal hyperexcitability assays	Sala et al. (2018)
Chemogenomic & Phenotypic Screening	Use of annotated compound libraries to identify phenotypic changes and infer mechanisms of action	• Drug target validation • Pathway deconvolution	Grafton et al. (2021)
Organotypic & Co-Culture Systems	3D organoid and microfluidic platforms mimicking tissue architecture and cell-cell interactions	• Neuro-muscular interaction studies • Disease modeling	Huh et al. (2010)
Omics Integration & Biomarker Discovery	Transcriptomic, proteomic, and biomarker analysis for mechanistic insight and precision therapy development	• Biomarker discovery • Patient stratification	Volpato et al. (2018)

### 3.3 The future of regenerative medicine: advancements and bottlenecks

Regenerative medicine has advanced significantly since the first derivation of hESCs in 1998 and the generation of hiPSCs in 2007. These cell types offer the ability to repair or replace damaged tissues, with hiPSCs providing additional advantages due to their patient-specific origin and ethical acceptability. The integration of hiPSC technology with precise genome editing and 3D organoid culture has expanded the potential for developing personalized cell-based therapies. Despite these breakthroughs, several critical challenges hinder clinical translation. Long-term culture of pluripotent cells can lead to tumorigenicity and genetic instability, necessitating rigorous quality control measures. hiPSC-derived therapeutic products must be evaluated for genomic integrity, purity, and sterility under cGMP (Liang et al., 2013). These requirements include the use of xeno-free, chemically defined media and the routine verification of normal karyotypes and pluripotency markers. Producing cell therapy products that comply with GMP standards can cost more than $800,000 per application. In addition, the cost to generate a single iPSC cell line can be as high as $20,000 (or $0.1 per well in a 96-well plate) using currently most selected substrates like Matrigel and Synthemax (Shi et al., 2017). Additional hurdles include establishing effective immunological tolerance, especially for hESC-based interventions. Addressing these technical, regulatory, and economic barriers is vital to unlock the full therapeutic promise of hPSCs in clinical regenerative medicine.

## 4 Current applications of hiPSC technology in toxicity screening

### 4.1 Application of hiPSC technology in toxicity screening

Traditional toxicity screening has long relied on animal models and immortalized cell lines, which often fail to predict accurate human responses to drugs and environmental toxins. This limitation has contributed to the high failure rates in drug development, with approximately 30% of potential therapeutics terminated due to unforeseen toxicities, particularly cardiotoxicity (Fritsche et al., 2021). This high attrition rate is largely attributed to the poor predictive capacity of conventional preclinical models, which fail to forecast human-specific toxicological outcomes reliably. The emergence of hiPSC technology has offered a transformative solution to these challenges by providing scalable, human-relevant cellular models for toxicity assessment. hiPSC-derived cells offer several advantages over conventional screening methods, including their human origin, ability to differentiate into disease-relevant cell types, and capacity to model patient-specific responses (Fritsche et al., 2020). The technology enables the development of predictive assays that can identify safety concerns early in the drug development process, potentially reducing costly late-stage failures.

Table 13 summarizes the advantages and limitations of both conventional and computational preclinical models. Conventional models each add value yet leave key translational gaps. Cancer cell lines are scalable and low-cost for target-based assays but drift genomically and lack tissue specificity and human predictivity (Trastulla et al., 2022). Primary human cells benchmark physiology but are short-lived, donor-variable, and hard to scale (Bulutoglu et al., 2020). Animal models provide systemic biology and PK/PD context yet face interspecies differences, ethics, and high cost/time (Yang and Papoian, 2018). *Ex vivo* tissues preserve native architecture but have short viability and low throughput (Jin et al., 2021). Two-dimensional cultures are reproducible but are missing matrix, mechanical, and paracrine cues needed for microenvironment fidelity (Ware et al., 2015). *In silico* approaches are rapid and inexpensive but depend on high-quality training data, require experimental validation, and have limited dynamical realism (Fritsche et al., 2020). Human iPSC-derived models address these limitations by generating renewable, patient-specific, lineage-accurate tissues; enabling isogenic controls and population genetics; and supporting advanced formats— organoids and micro-physiological chips— that restore microenvironmental mechanics, multicellular interactions, and cross-tissue crosstalk, thereby improving mechanistic insight and human predictivity.

**TABLE 13:** Advantages and limitations of conventional and computational preclinical models.

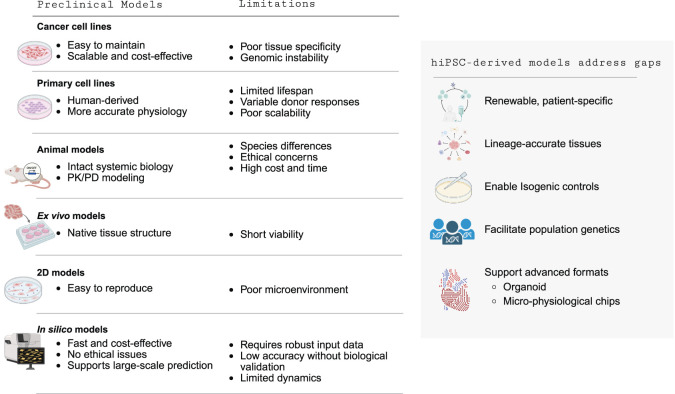

### 4.2 hiPSC in toxicology: advancing beyond traditional models

hiPSC-based toxicity screening offers compelling advantages over traditional methods, addressing fundamental limitations in current safety assessment paradigms. The human relevance of iPSC-derived models represents a critical breakthrough, overcoming species differences inherent in animal models that often fail to predict human responses (Fritsche et al., 2020). Mouse models, for instance, poorly mimic human inflammatory diseases and drug responses, leading to the need for human-based systems (Seok et al., 2013). hiPSCs can be differentiated into diverse cell types, including hepatocytes, cardiomyocytes, neurons, and other disease-relevant cells, enabling comprehensive toxicity assessment across multiple organ systems. The technology’s capacity for patient-specific modeling allows personalized toxicity testing and pharmacogenomics studies, enabling researchers to assess how genetic variations influence individual susceptibility to toxic compounds (Yildirim et al., 2025). This approach facilitates disease modeling by testing compounds in disease-affected cell lines, demonstrating how pathological conditions may alter toxicity profiles. hiPSC platforms have high-throughput potential and are compatible with automation and multi-well formats for large-scale screening applications (Tristan et al., 2021). Additionally, these systems offer significant ethical advantages by reducing animal usage, aligning with the 3Rs principle (replacement, reduction, and refinement), and supporting regulatory initiatives like the FDA Modernization Act 2.0 (Yildirim et al., 2025; Tannenbaum and Bennett, 2015).

### 4.3 Integrated toxicity screening using iPSC-derived human cells

Human iPSC-derived cell types are increasingly utilized across a broad range of toxicological contexts, providing organ-specific models that enhance the detection and characterization of adverse effects.

**FIGURE 2:**
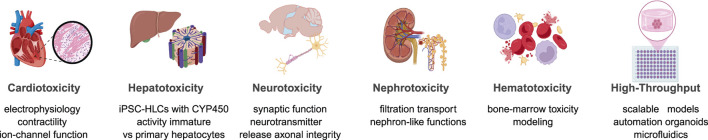
Current applications of iPSC technology in toxicity screening.

Figure 2 summarizes the current applications of hiPSC-derived cells in modeling toxicity across various organ systems. Specifically, hiPSC cardiomyocytes quantify proarrhythmic risk, including QT prolongation, hERG blockade, contractility deficits, and drug-induced arrhythmias (Visone et al., 2023). Moreover, hiPSC hepatocyte-like cells enable DILI assessment by capturing mitochondrial dysfunction, bile-acid transport defects, and broader metabolic toxicities (Tamargo-Rubio et al., 2023). Likewise, neural hPSC models (neurons and astrocytes) reveal seizure liability, neurodevelopmental perturbations, neurodegeneration, and synapto-toxicity (Tukker et al., 2019). In addition, kidney models composed of proximal tubule and podocyte lineages resolve filtration and transporter-mediated injuries typical of agents such as cisplatin and aminoglycosides (Lawrence et al., 2022). Similarly, hematopoietic progenitor systems detect marrow suppression, genotoxicity, and cytopenias (Fransen and Leonard, 2023). Finally, integrated platforms that combine organoids, micro-physiological chips, automation, and high-content phenotyping extend these assays to scalable safety pharmacology and compound prioritization (Vandana et al., 2023). Collectively, these human-relevant models increase mechanistic resolution and improve translational decision-making.

The power of hiPSC-based toxicity screening is significantly enhanced by integrating advanced analytical technologies that enable detailed and high-throughput assessment of cellular responses.

Table 14 summarizes key technologies, their applications, and associated methodological features in current hiPSC-based toxicology platforms. High-content imaging quantifies morphology, apoptosis, and mitochondrial integrity in hiPSC-derived cardiomyocytes, hepatocytes, and neurons, enabling scalable phenotypic screening (Tolosa et al., 2015). Microelectrode arrays and calcium assays in hiPSC-cardiomyocytes provide electrophysiological endpoints aligned with pro-arrhythmia risk assessment and CiPA frameworks (Vandana et al., 2023). Brain, liver, and kidney organoids supply tissue-level architecture and paracrine signaling for systems-level toxicity readouts, albeit with variability that requires rigorous QC (Grimm et al., 2015). Multi-omics layers (transcriptomics, proteomics, metabolomics) map mechanism-of-action and furnish candidate biomarkers for stratification and translation (Nabi et al., 2022). CRISPR-engineered isogenic hiPSC lines resolve gene–environment and pharmacogenomic interactions, strengthening causal inference and target validation (Kim et al., 2020; Sayed et al., 2020). Together, these integrated modalities deliver human-relevant, multiparametric endpoints that improve predictive validity and support regulatory-grade decision making.

**TABLE 14:** Integrated technologies supporting hiPSC-based toxicity screening: applications, capabilities, and limitations.

Technology	Application	Cell type used	Toxicity Endpoint Measured	Validation status	Limitations	Refs
High-Content Imaging	Phenotypic screening; quantifying injury or death	Various (cardiomyocytes, hepatocytes, neurons)	Cell morphology, apoptosis, mitochondrial dysfunction	Widely used; not yet regulatory standard	Requires specialized imaging systems and analysis tools	Tolosa et al. (2015)
Microelectrode Arrays (MEAs)	Cardiotoxicity detection; arrhythmia modeling	iPSC-cardiomyocytes	Field potential duration, beat rate, arrhythmia	Integrated into FDA, CiPA	Low scalability; limited to electrophysiology	Grimm et al. (2015)
Organoid Models	Organ-specific toxicity testing	Brain, liver, kidney organoids	Tissue-level injury, cell-cell signaling	OECD consideration for DART	Variability; complex culture and analysis	Nabi et al. (2022)
Omics (Transcriptomics/Metabolomics)	Mechanistic toxicology; biomarker discovery	Various iPSC-derived cells	Gene/protein/metabolite changes	Exploratory; research-focused	Costly; requires bioinformatics	Kim et al. (2020)
CRISPR/Cas9 Editing	Gene-toxicity interaction; pharmacogenomics	Isogenic iPSC lines	Genetic sensitivity, mutation impact	Widely used in research	Off-target effects; needs validation	Peng et al. (2023)

### 4.4 Future directions in hiPSC-based toxicity screening

The field of hiPSC-based toxicity screening is undergoing rapid transformation, driven by advances that aim to enhance physiological relevance, scalability, and predictive accuracy. Efforts are focused on developing mature, multicellular organoid systems that better replicate adult tissue functionality through strategies such as mechanical stimulation, extended culture, and co-differentiation. Concurrently, the integration of hiPSC-derived tissues with organ-on-a-chip platforms is enabling dynamic modeling of tissue-tissue interfaces, fluidic flow, and multi-organ interactions—key for capturing systemic toxicity (Park et al., 2019). AI is increasingly applied to extract meaningful patterns from high-dimensional datasets, including omics, imaging, and electrophysiology, thereby improving early toxicity prediction (Vo et al., 2024; Tran et al., 2023). Furthermore, the expansion of hiPSC models into environmental and precision toxicology is enabling the study of gene-environment interactions and personalized risk profiles.

**FIGURE 3:**
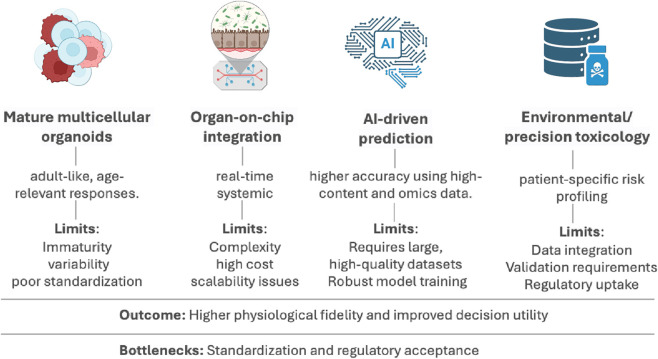
Advancing iPSC-based toxicity screening-technologies, applications, and translational considerations.

These innovations (Figure 3) collectively position hiPSC-based systems as next-generation platforms for mechanistic toxicology, regulatory testing, and individualized safety assessment. Mature multicellular organoids, organ-on-chip integration, AI-driven prediction, and environmental/precision toxicology are converging to increase physiological fidelity and decision utility. Organoid maturation by prolonged culture, defined mechanical cues, and strategic co-culture improves adult-like and age-relevant responses, yet immaturity, variability, and limited standardization persist (Fritsche et al., 2021). Coupling hiPSC-derived tissues to microfluidic chips enables controlled flow, tissue–tissue interfaces, and multi-organ coupling for dynamic, real-time readouts; however, complexity, cost, and scalability on these systems remain as barriers (Ahmed and Moreira Teixeira, 2022). Machine-learning models that mine high-content imaging, electrophysiology, and multi-omics can reveal subtle injury signatures and improve prediction, but they require large, high-quality datasets, rigorous model development and validation (Tran et al., 2023). Patient-specific hiPSC models support quantification of genotype–exposure interactions for individualized risk assessment and public-health protection; robust data integration, external validation, and regulatory uptake are still needed to validate this individual models (Chandy et al., 2022). Collectively, these trajectories position hiPSC platforms for next-generation mechanistic toxicology, regulatory testing, and individualized safety assessment, contingent on harmonized methods and regulatory alignment (Tran et al., 2023; Ahmed and Moreira Teixeira, 2022; Chandy et al., 2022; Fritsche et al., 2021).

### 4.5 Barriers to implementation of hiPSC-based toxicology platforms

**FIGURE 4:**
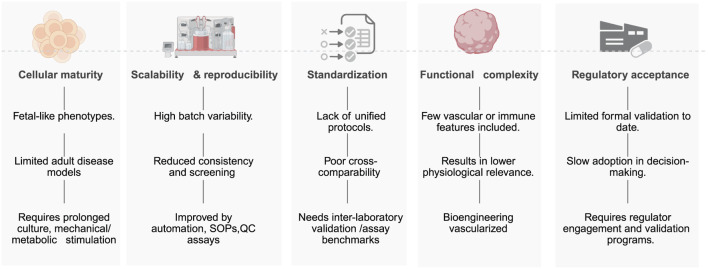
Barriers to implementation in iPSC-Based Toxicity Screening.

Figure 4 illustrates barriers to implementing hiPSC-based toxicology platforms, which include persistent cellular immaturity across lineages—such as low IK1 and glycolytic bias in cardiomyocytes (Yang et al., 2014), fetal CYP profiles in hepatocytes (Gurevich et al., 2020), and incomplete synaptic/myelination features in neural cultures (James et al., 2021), which limits modeling of adult and chronic toxicities. Other barriers include: limited scalability and reproducibility driven by donor, clone, and batch effects and by matrix, media, and passage-dependent drift, reducing inter-laboratory concordance and HTS readiness (Volpato and Webber, 2020); lack of harmonized protocols, standardized endpoints, and qualified QC materials, which impedes cross-study comparison and assay qualification (OECD, 2018); restricted functional complexity due to absent vasculature, immune components, and organ-level microanatomy (e.g., sinusoid, glomerulus, neurovascular unit), diminishing physiological fidelity (Yip et al., 2023); and lagging regulatory acceptance because contexts of use, reference compound sets, performance standards, and formal validation remain incomplete despite enabling policies (e.g., FDA Modernization Act 2.0) (Sewell et al., 2024). Active mitigation actions includes maturation strategies (prolonged culture, metabolic/electrical/mechanical conditioning, co-culture), automated standardized SOPs with in-process QC and reference panels, interlaboratory ring trials, and bioengineered, vascularized, immune-competent, 3D, and micro-physiological systems advanced with regulators (Sewell et al., 2024). Addressing these bottlenecks will transition hiPSC assays from research tools to validated human-relevant tests that support regulatory safety decision-making.

## 5 Conclusion

The advent of hiPSCs represents a revolutionary advancement in the field of preclinical safety assessment and pharmacological research. By enabling the reprogramming of human somatic cells into pluripotent stem cells, hiPSCs provide an ethically sound, renewable, and genetically matched cellular resource for drug discovery, toxicity testing, and disease modeling (Kleiman and Engle, 2021). This innovation significantly improves the predictive accuracy of organ-specific toxicities and individual patient responses, surpassing traditional animal models, which frequently fail to replicate human-specific biological processes and adverse reactions accurately (Tran et al., 2023).

Current hiPSC-based models have notably enhanced the efficiency and accuracy of high-throughput drug screening by offering the capability to differentiate into diverse cell lineages, including cardiomyocytes, hepatocytes, neurons, endothelial cells, and renal cells (Tristan et al., 2021). These cell models facilitate detailed mechanistic investigations and more precise efficacy and safety evaluations, thus streamlining the drug development pipeline and substantially reducing costs and development timelines. Particularly in toxicology, hiPSC-derived cellular systems have demonstrated their potential to identify toxic effects missed by conventional animal studies, thereby significantly reducing attrition rates in later stages of clinical trials (Olson et al., 2000).

Moreover, the integration of hiPSC technology with advanced complementary platforms such as 3D organoids, microfluidic organ-on-a-chip systems, and AI-driven analytics has dramatically broadened the scope and depth of preclinical studies (Yildirim et al., 2025). These technological synergies enable a closer simulation of complex human physiological environments, improved modeling of dynamic tissue-tissue interactions, and more accurate prediction of drug metabolism, pharmacokinetics, and pharmacodynamics (Jin et al., 2021). Such advancements are crucial for improving precision medicine and personalizing therapeutic interventions, highlighting the transformative potential of iPSC technology in both pharmaceutical innovation and personalized patient care.

Nevertheless, despite these substantial advancements, the full translational and clinical potential of hiPSC-based platforms faces persistent challenges. Key issues include inherent variability between hiPSC lines due to differences in genetic background, reprogramming methods, and clonal selection processes. Additionally, standardized differentiation and assay protocols remain limited, complicating cross-study comparisons and regulatory acceptance. Another critical obstacle is the immature phenotype of hiPSC-derived cells, which often exhibit fetal or neonatal characteristics rather than fully mature adult functionalities, thereby limiting their predictive capabilities for chronic toxicities and adult-onset diseases (Volpato et al., 2018; Yang et al., 2014; OECD, 2018; Yip et al., 2023; Sewell et al., 2024).

Addressing these challenges requires ongoing interdisciplinary collaboration, robust quality control measures, and the development of standardized, reproducible methodologies. Advances in automation, biomanufacturing, and real-time monitoring technologies are anticipated to alleviate issues related to scalability and reproducibility, further facilitating the broader adoption of hiPSC-derived platforms across academic and industrial research environments (Fritsche et al., 2021).

Regulatory frameworks must also evolve concurrently to fully integrate hiPSC-based models into standard drug safety assessment practices. Encouragingly, regulatory agencies like the FDA have already begun initiatives, such as the FDA Modernization Act 2.0, which advocate for the inclusion of advanced human-relevant methods to reduce animal testing (Yildirim et al., 2025; Ahmed et al., 2023). This regulatory support underscores the growing recognition of hiPSC technology as a scientifically credible and ethically preferable alternative within toxicology and drug discovery.

Building on this regulatory momentum, there is broad consensus that human-relevant, xeno-free alternatives are needed to address the poor translation of animal models and to operationalize the 3Rs (Tannenbaum and Bennett, 2015; Tanner, 2018). Accordingly, xeno-free hPSC and organoid systems are prioritized to reduce biosafety and variability and to meet GMP expectations for clinical translation (Rodríguez-Pizà et al., 2010). International bodies have established acceptance pathways for non-animal testing methods. Organization for Economic Co-operation and Development (OECD) Test Guidelines now include human-relevant alternatives, while European Union Reference Laboratory (EURL) -Reference Laboratory for Alternatives to Animal Testing (ECVAM) provides validation frameworks (Stephens et al., 2013). Collectively, these positions justify and accelerate adoption of xeno-free, human-based platforms for preclinical safety assessment and drug discovery.

In summary, hiPSC technology has become an indispensable asset in modern pharmacological research and toxicological evaluation, poised to significantly transform preclinical drug testing paradigms. Continued advancements in differentiation protocols, standardization efforts, and regulatory harmonization are vital to overcoming existing barriers and fully realizing the transformative potential of hiPSCs. Ultimately, the integration of hiPSC technology with emerging advanced technologies promises safer, more effective, and more personalized medical therapies, profoundly impacting public health outcomes and revolutionizing the pharmaceutical industry.
